# Prevalence of dental caries and influence factors among students in Beijing: A cross-sectional study

**DOI:** 10.1371/journal.pone.0322694

**Published:** 2025-04-29

**Authors:** Youhan Wen, Yu Liu, Ning Ning, Huiyu Xu, Jingshu Zhang, Hai Zhao, Ran Qin, Lu Wang, Xin Guo, Liyuan Tao

**Affiliations:** 1 Research Center of Clinical Epidemiology, Peking University Third Hospital, Beijing, China; 2 Department of Global Public Health,Karolinska Institutet, Stockholm, Sweden; 3 Institute of Basic Research in Clinical Medicine, China Academy of Chinese Medical Sciences, Beijing, China; 4 Key Laboratory of Environmental Stress and Chronic Disease Control & Prevention, Ministry of Education, China Medical University, Shenyang Liaoning, China; 5 Department of Biostatistics and Epidemiology, School of Public Health, China Medical University, Shenyang, Liaoning, China; 6 Institute of School Health, Beijing Center for Disease Prevention and Control, Beijing, China; 7 School of Public Health, Capital Medical University, Beijing, China; 8 Medical Examination Centre, Peking University Third Hospital, Beijing, China; Shahid Beheshti University of Medical Sciences School of Dentistry, ISLAMIC REPUBLIC OF IRAN

## Abstract

**Background:**

The burden of dental caries continues to be substantial worldwide, significantly affecting adolescents and children’s growth and quality of life. This study aimed to explore the epidemiological characteristics of dental caries and examine influencing factors that contribute to caries among children and adolescents in Beijing.

**Methods:**

A total of 21,403 students (average age 12.8 ± 2.5 years old) were included in this cross-sectional survey. The DMFT/dmft index (decayed (D), missing (M), and filled (F) teeth) was used for screening. Multistage stratified random cluster sampling methods were conducted in primary and secondary schools in Beijing in 2021. Data were collected using questionnaires and assessed using a logistic regression analysis. Statistical significance was set at P < 0.05.

**Result:**

The overall prevalence of caries was 40.5% (95% CI: 39.8–41.1%). The mean score of DMFT was 1.31 ± 2.11, and the mean score of dmft was 0.39 ± 1.24. Among participants, girls, students in lower grades, those of younger ages, and those with lower BMI levels were more likely to experience dental caries. A multivariate logistic regression analysis identified the following variables as predictors of an increased risk for caries experience: residing in boarding school (odds ratio [OR]=1.15); coming from a rural area (OR = 2.55); frequent consumption of sugary beverages (ORmax = 1.23), and fresh fruit (ORmax = 1.35); breakfast irregularity (ORmax = 1.22); infrequent tooth brushing (ORmax = 1.16); and longer sleep duration (ORmax = 1.30).

**Conclusions:**

Dental caries, widely prevalent among children and adolescents in Beijing, is associated with dietary habits, hygiene practices, and sleep patterns. To effectively mitigate its burden, prevention strategies targeting these identified risk factors should be prioritized.

## Introduction

Dental caries, recognized as the most prevalent noncommunicable disease globally, is a leading cause of hospitalization among children in some high-income countries [1]. Dental caries impose significant economic burdens and adversely affect the health of children and adolescents, impacting an estimated 2.3 billion adults and over 530 million children across various regions of the world [2,3]. In China, the fourth national survey on oral health epidemiology in 2015 revealed that 34.5% of 12-year-old children have caries in their permanent teeth, marking an increase of 7.8 percentage points from 10 years ago [4]. DMFT/dmft represents the total number of Decayed, Missing due to caries, and Filled Teeth in the permanent teeth/primary teeth [5]. This indicator belongs to a set of indicators designed to assess the status of dental caries [5].

Previous literature has identified various risk factors for dental caries in children, which were conceptually categorized as related to socio-demographic characteristics, dental hygiene, diet, and dental services [6]. Regarding primary teeth, dental caries was found to be inversely associated with BMI-for-age [7]. Additionally, nutritional status and dietary habits significantly impacted the development and integrity of the oral cavity and the progression of oral diseases [8]. It was also found that consuming fruit and vegetables among adolescents was associated with dental erosion [9]. Furthermore, the WHO indicated that lower sugary drink intake improves nutrition and fewer people suffering from tooth decay [10]. Growing evidence has confirmed that using topical fluoride and general prophylaxis can effectively manage untreated caries [11].

In Beijing, a mega-city with a vast population, it was meaningful to measure and study the condition of common diseases in adolescents and children. We aimed to investigate the prevalence of dental caries among children and adolescents in Beijing, identify the main factors affecting their health, and fulfill monitoring tasks to take targeted intervention measures.

## Materials and methods

### Study design

This study targeted children and adolescents aged 8–20 in Beijing as the study participants. The research sample was drawn from health surveys conducted through a collaborative effort involving Beijing Center for Disease Control and Prevention, Beijing Municipal Health Commission, and Beijing Municipal Education Commission. Recruitment began on September 8^th^,2021 and ended on November 10^th^, 2021.

### Survey sampling

A multi-stage stratified random cluster survey was conducted to select participants who were representative of students of all districts of Beijing [12]. In the first stage, schools across Beijing were categorized into three groups based on the student population of the districts: those with more than 90,000 students were categorized as type one, those with 60,000–90,000 students as type two, and those with fewer than 60,000 students as type three. In the second stage, 28 schools were randomly selected from type one districts (including 10 primary schools, 10 junior high schools, and 8 senior high schools), 38 schools from type two districts (including 14 primary schools, 14 junior high schools, 10 senior high schools,), and 26 schools from type three districts (including 10 primary schools, 10 junior high schools, 6 senior high schools). In the third stage, at least 80 students were randomly selected from each grade in every school. For primary schools, which have 6 grades, this resulted in a minimum of 480 students per school, while for junior high and senior high schools, which have 3 grades, this resulted in a minimum of 240 students per school. Students were chosen using whole cluster sampling with classes as the smallest sampling unit. The total number of students randomly selected for this study was at least 30,240.

This study focused on identifying factors influencing dental caries among the student population based on data collected through self-administered questionnaires. Participants and their legal guardians were required to write informed consent forms. Students suffering from severe physical or psychological conditions or impairments, who were unable or unwilling to complete the examination and questionnaire were excluded. Since students from grades 1–3 in primary schools have limited literacy, they were not surveyed. After excluding primary school students from grades 1–3, the study included 22,080 participants. Consequently, the study included 21,403 students. The non-response rate was 3.07%. Losses were attributed to several factors: 185 participants refused dental examinations, 371 had missing data in their questionnaires, and 121 were absent on the days scheduled for the dental examinations (S1 Fig).

### Ethical consideration

The study was approved by the Ethical Committee of the Peking University Third Hospital (IRB00006761-M2020319) and conducted according to the guidelines of the Declaration of Helsinki. Written informed consent was obtained from participating students, along with their parents or guardians.

### Quality control

48 professional oral physicians were health professionals and technicians who received pre-service training before October 2020. Cohen’s kappa statistics, assessing the consistency of inter- and intra-examiner, consistently exceeded 0.75. Professional oral physicians were proficient in detection methods and were qualified by the expert group to participate in the survey. Each professional testing group had specialized quality control members responsible for checking the quality of the tests. It included face-to-face verification of examinees’ information and the random re-evaluation of 5% of participants during daily testing to ensure the accuracy of the results. If the re-evaluation results did not pass, all examination results from that dentist were discarded, and other oral physicians were selected to re-examine the participants. The self-administered questionnaires filled out by the study participants were checked by 180 professionals upon submission.

### Questionnaire survey

In this study, we used a self-administered questionnaire to investigate the participants’ health status and lifestyle habits. The questionnaire covered basic information, diet and exercise behaviors, and daily health habits. For dietary and exercise behaviors, consuming sugary drinks and fried foods in the past 7 days was categorized into three levels: never, less than once per day, and one or more times per day. Fresh fruit intake and vegetable intake over the past 7 days were divided into four levels: never, less than once per day, once per day, and two or more times per day. Having breakfast was classified into three levels: every day, occasionally, and never. In the daily health behaviors module, tooth brushing habits were classified into four levels: brushing both morning and evening“, brushing only in the morning, brushing only in the evening, and brushing occasionally or never. The use of fluoridated toothpaste was categorized as yes, no, don’t know. In addition, the average sleep time needs to be filled out by the participants (S1 Appendix).

### Clinical examination

All examinations were done at school and by a licensed dentist from the hospital’s dental department. Each participant in the selected regions underwent an oral health examination conducted by a trained and licensed dentist following WHO guidelines [13]. The examination utilized portable equipment, including a dental chair, external light source, flat dental mirror, and the community periodontal index probe (CPI probe/ WHO); participants are orientated to the light source, following WHO clinical criteria and visual assessment methods [13]. The caries was documented as present when a lesion in a pit, fissure, or on a smooth tooth surface exhibits a clear cavity, undermined enamel, or a noticeably softened floor or wall [13]. The DMFT/dmft index was used as the metric for assessing caries experience in our evaluation. Teeth that were decayed (d for primary teeth, D for permanent teeth), missing (m for primary teeth, M for permanent teeth), or filled (f for primary teeth, F for permanent teeth) were calculated into the DMFT/dmft index for mixed dentition, following WHO guidelines [13].

After the clinical examination, the doctor recorded the results on the medical record form. DMFT and dmft were used to represent the health status of primary and permanent teeth, respectively. If the amount of caries in either is greater than 0, it was defined as caries in primary or permanent teeth. Additionally, if the caries was present in either primary or permanent teeth, the participants were defined as having caries.

### Statistical analysis

After data aggregation, each district consolidated its databases and forwarded them to the School Health Department of the Beijing Municipal Center for Disease Control and Prevention. All data were gathered from the questionnaires and oral health evaluation forms. EpiData Version 3.1 (EpiData Association, http://www.epidata.dk, Odense, Denmark) was utilized to enter the data. To ensure data consistency and accuracy, each item was verified at least three times during the entry process.

After data cleaning was completed, the data were locked. For continuous variables, the Kolmogorov-Smirnov test was used for the normality test. Continuous variables that conformed to a normal distribution were described as mean ± standard deviation and compared between groups using t-tests or analysis of variance (ANOVA). Categorical data were described using n (%) and compared between groups using chi-square tests. Multivariate logistic regression analysis was conducted to ascertain the predominant predictors of caries among variables such as age, sex, and brushing habits. All statistical operations were conducted using SPSS 26.0 software, a *P* value of < 0.05 was considered statistically significant.

## Result

A total of 21,403 student study participants ranging from 4th grade of primary school to high school were included in this study, of which 10,882 (50.84%) were males and 10,521 (49.16%) were females. The average age of the participants was 12.80 ± 2.51 years, and their mean BMI was 21.55 ± 4.86 kg/m^2^. Dental caries occurred in 8,659 of the total study population, indicating a prevalence rate of 40.5% (primary and permanent teeth) (95% CI = 39.8%-41.1%). The prevalence of dental caries was notably higher in girls than in boys (42.43% vs 38.55%). Our study also revealed that the prevalence of dental caries decreased as school stages developed, from primary school to junior school to high school (41.79% vs. 41.57% vs. 36.95%), with these differences being statistically significant (*P* < 0.001). Students from rural areas have a higher prevalence of dental caries compared to those from urban areas (48.56% vs 26.44%). In conclusion, students with dental caries were younger, had a lower BMI, and were more likely to be girls. They consumed sugary beverages and fresh fruit more frequently and used fluoride toothpaste less often. Additionally, they averaged more hours of sleep compared to students without dental caries (Table 1).

**Table 1:** Baseline characteristics of the study population in Beijing.

Variable	Total	Caries (primary teeth or permanent teeth)		p
		with	without	
Age	12.80 ± 2.51	12.65 ± 2.53	12.90 ± 2.49	<0.001
BMI	21.55 ± 4.86	21.41 ± 4.87	21.64 ± 4.85	0.001
Ethnic				0.059
Han	19165(89.54)	7795(40.67)	11370(59.33)	
Other	2238(10.46)	864(38.61)	1374(61.39)	
Sex				<0.001
Male	10882(50.84)	4195(38.55)	6687(61.45)	
Female	10521(49.16)	4464(42.43)	6057(57.57)	
Areas				<0.001
Urban area	7840(36.63)	2073(26.44)	5767(73.56)	
Rural area	13563(63.37)	6586(48.56)	6977(51.44)	
Education stages				<0.001
Primary school 4–6	7985(37.31)	3337(41.79)	4648(58.21)	
Junior high school	7868(36.76)	3271(41.57)	4597(58.43)	
Senior high school	5550(25.93)	2051(36.95)	3499(63.05)	
Boarding school				<0.001
Yes	3035(14.18)	1390(45.80)	1645(54.20)	
No	18368(85.82)	7269(39.57)	11099(60.43)	
Chronic disease history				0.203
No	20829(97.32)	8412(40.39)	12417(59.61)	
Yes	574(2.68)	247(43.03)	327(56.97)	
Drinking sugary beverages (past 7 days)				0.001
Never	6646(31.05)	2588(38.94)	4058(61.06)	
Less than 1 time per day	13527(63.20)	5532(40.90)	7995(59.10)	
1 or more times per day	1230(5.75)	539(43.82)	691(56.18)	
Eating fried food (past 7 days)				0.001
Never	5465(25.53)	2205(40.35)	3260(59.65)	
Less than 1 time per day	15035(70.25)	6034(40.13)	9001(59.87)	
1 or more times per day	903(4.22)	420(46.51)	483(53.49)	
Eating fresh fruit (past 7 days)				0.032
Never	660(3.08)	241(36.52)	419(63.48)	
Less than 1 time per day	3555(16.61)	1467(41.27)	2088(58.73)	
Once a day	10168(47.51)	4052(39.85)	6116(60.15)	
2 or more times per day	7020(32.80)	2899(41.30)	4121(58.70)	
Eating vegetables (past 7 days)				<0.001
Never	350(1.64)	150(42.86)	200(57.14)	
Less than 1 time per day	1545(7.22)	701(45.37)	844(54.63)	
Once a day	5724(26.74)	2421(42.30)	3303(57.70)	
2 or more times per day	13784(64.40)	5387(39.08)	8397(60.92)	
Having breakfast (past 7 days)				<0.001
Every day	17560(82.04)	6919(39.40)	10641(60.60)	
Occasionally	3234(15.11)	1473(45.55)	1761(54.45)	
Never	609(2.85)	267(43.84)	342(56.16)	
Tooth brushing habit				<0.001
Morning and evening	17051(79.67)	6823(40.02)	10228(59.98)	
Only morning	2109(9.85)	922(43.72)	1187(56.28)	
Only evening	1732(8.09)	682(39.38)	1050(60.62)	
Occasionally or never	511(2.39)	232(45.40)	279(54.60)	
Using fluoride toothpaste				<0.001
Yes	6118(28.58)	2332(38.12)	3786(61.88)	
No	3067(14.33)	1264(41.21)	1803(58.79)	
Don’t know	12218(57.09)	5063(41.44)	7155(58.56)	
Average daily sleep duration				<0.001
< 8h	7698(35.97)	2913(37.84)	4785(62.16)	
8-10h	13313(62.20)	5562(41.78)	7751(58.22)	
> 10h	392(1.83)	184(46.94)	208(53.06)	
Days of exercise per week (over 60 minutes)				0.063
0 days	1467(6.85)	613(41.79)	854(58.21)	
1 days	1612(7.53)	647(40.14)	965(59.86)	
2 days	2506(11.71)	1021(40.74)	1485(59.26)	
3 days	2951(13.79)	1127(38.19)	1824(61.81)	
4 days	2057(9.61)	820(39.86)	1237(60.14)	
5 days	4126(19.28)	1694(41.06)	2432(58.94)	
6 days	1354(6.33)	521(38.48)	833(61.52)	
7 days	5330(24.90)	2216(41.58)	3114(58.42)	

The mean score for dmft (decayed, missing, filled teeth in primary teeth) was recorded as 0.39 ± 1.24. This score decreased with increasing BMI. Girls had lower dmft scores than boys. Additionally, fresh fruit consumption was correlated with higher dmft scores, whereas the utilization of fluoride toothpaste contributed to lower dmft scores (Table 2). The mean score for DMFT was 1.31 ± 2.11. Higher DMFT scores were observed with increasing age and BMI, and girls had higher DMFT scores than boys. Additionally, higher DMFT scores were observed among those consuming sugary beverages and fried foods, whereas lower DMFT scores were observed among those who regularly had breakfast and used fluoride toothpaste (Table 2).

**Table 2:** DMFT/dmft situation of research participants with different characteristics.

	DMFT+dmft		dmft (primary teeth)		DMFT (permanent teeth)	
	Mean±std	p	Mean±std	p	Mean±std	p
Age		0.003		<0.001		<0.001
8-12	1.67 ± 2.34		0.80 ± 1.69		0.87 ± 1.54	
12-15	1.70 ± 2.40		0.03 ± 0.21		1.68 ± 2.38	
> 15	1.82 ± 2.56		0.01 ± 0.09		1.81 ± 2.56	
BMI group		<0.001		<0.001		<0.001
< 18	1.92 ± 2.55		0.79 ± 1.73		1.13 ± 1.90	
18-24	1.69 ± 2.38		0.33 ± 1.13		1.37 ± 2.15	
24-28	1.55 ± 2.28		0.20 ± 0.80		1.35 ± 2.17	
>=28	1.51 ± 2.26		0.08 ± 0.47		1.43 ± 2.24	
Ethnic		<0.001		0.064		<0.001
Han	1.73 ± 2.43		0.40 ± 1.26		1.33 ± 2.13	
Other	1.50 ± 2.18		0.35 ± 1.14		1.15 ± 1.88	
Sex		<0.001		<0.001		<0.001
Male	1.52 ± 2.30		0.43 ± 1.33		1.10 ± 1.92	
Female	1.90 ± 2.49		0.36 ± 1.15		1.53 ± 2.27	
Areas		<0.001		<0.001		<0.001
Urban area	1.23 ± 1.96		0.25 ± 0.97		0.98 ± 1.77	
Rural area	1.98 ± 2.58		0.48 ± 1.37		1.50 ± 2.26	
School level		<0.001		<0.001		<0.001
Primary school (4–6)	1.75 ± 2.41		1.00 ± 1.86		0.75 ± 1.39	
Junior high school	1.62 ± 2.29		0.05 ± 0.33		1.57 ± 2.26	
Senior high school	1.77 ± 2.53		0.01 ± 0.09		1.76 ± 2.53	
Board at school		0.455		<0.001		<0.001
Yes	1.74 ± 2.38		0.08 ± 0.54		1.65 ± 2.35	
No	1.70 ± 2.40		0.45 ± 1.32		1.26 ± 2.06	
Chronic medical History		0.074		0.001		<0.001
No	1.70 ± 2.39		0.40 ± 1.25		1.30 ± 2.09	
Yes	1.88 ± 2.70		0.22 ± 0.91		1.67 ± 2.59	
Drinking sugary beverages (past 7 days)		<0.001		<0.001		<0.001
Never	1.61 ± 2.33		0.46 ± 1.31		1.16 ± 1.96	
Less than1times (per day)	1.74 ± 2.42		0.37 ± 1.22		1.37 ± 2.16	
1 or more times (per day)	1.87 ± 2.52		0.34 ± 1.16		1.52 ± 2.29	
Eating fried food (past 7 days)		<0.001		<0.001		<0.001
Never	1.65 ± 2.34		0.48 ± 1.34		1.18 ± 1.93	
Less than 1 times (per day)	1.71 ± 2.41		0.37 ± 1.21		1.34 ± 2.15	
1 or more times (per day)	2.01 ± 2.55		0.38 ± 1.22		1.63 ± 2.34	
Eating fresh fruit (past 7 days)		<0.001		<0.001		<0.001
Never	1.36 ± 2.14		0.23 ± 0.93		1.13 ± 1.90	
Less than 1 time (per day)	1.71 ± 2.45		0.24 ± 0.98		1.47 ± 2.30	
Once a day	1.68 ± 2.38		0.34 ± 1.16		1.34 ± 2.13	
2 or more times (per day)	1.78 ± 2.43		0.56 ± 1.47		1.21 ± 1.99	
Eating vegetables (past 7 days)		<0.001		0.439		<0.001
Never	1.52 ± 2.21		0.32 ± 1.21		1.20 ± 1.84	
Less than 1 time (per day)	1.87 ± 2.54		0.43 ± 1.29		1.44 ± 2.26	
Once a day	1.83 ± 2.54		0.38 ± 1.25		1.44 ± 2.28	
2 or more times (per day)	1.64 ± 2.33		0.40 ± 1.24		1.25 ± 2.02	
Having breakfast (past 7 days)		<0.001		<0.001		<0.001
Every day	1.67 ± 2.36		0.43 ± 1.29		1.23 ± 2.03	
Occasionally	1.92 ± 2.60		0.25 ± 1.02		1.67 ± 2.45	
Never	1.76 ± 2.37		0.13 ± 0.66		1.63 ± 2.29	
Tooth brushing Habit		0.337		<0.001		0.772
Both morning and Evening	1.71 ± 2.40		0.41 ± 1.26		1.31 ± 2.10	
Only morning	1.62 ± 2.29		0.26 ± 1.03		1.36 ± 2.09	
Only evening	1.75 ± 2.55		0.43 ± 1.33		1.32 ± 2.27	
Occasionally or Never	1.71 ± 2.33		0.40 ± 1.29		1.31 ± 2.03	
Using fluoride toothpaste		<0.001		<0.001		<0.001
Yes	1.63 ± 2.32		0.49 ± 1.38		1.14 ± 1.92	
No	1.68 ± 2.42		0.56 ± 1.48		1.12 ± 1.96	
Do not know	1.75 ± 2.44		0.30 ± 1.09		1.45 ± 2.22	
Average daily sleep duration		0.171		<0.001		<0.001
< 8 h	1.72 ± 2.45		0.08 ± 0.56		1.64 ± 2.40	
8–10 h	1.69 ± 2.37		0.56 ± 1.45		1.13 ± 1.90	
> 10 h	1.90 ± 2.61		0.95 ± 1.91		0.95 ± 1.75	

The multivariate logistic regression analysis showed that the prevalence of caries decreased as age increased. Boys exhibited a lower prevalence of dental caries than girls (OR = 0.84, 95% CI = 0.79–0.89, *P* < 0.001). The lower grade level was associated with a higher prevalence of dental caries (ORmax = 1.41, 95% CI = 1.14–1.73, *P* = 0.001). Additionally, boarding school students had a higher risk of dental caries compared to non-boarding school students (OR = 1.15, 95% CI = 1.05–1.26, *P* = 0.003). Students from rural areas exhibit a stronger association with the occurrence of dental caries compared to urban areas (OR = 2.55, 95% CI = 2.40–2.72, *P < *0.001). Compared to never consuming sugary drinks, consuming them was positively correlated with the occurrence of dental caries (ORmax = 1.23, 95% CI = 1.07–1.42, *P* = 0.004). As the frequency of fruit consumption increased, the association between this risk factor and dental caries became stronger (ORmax = 1.35, 95% CI = 1.12–1.62, *P* = 0.001). Having breakfast irregularly (ORmax = 1.22, 95% CI = 1.05–1.42, *P* = 0.048) was associated with an increased prevalence of dental caries. Furthermore, longer sleep durations were associated with a higher risk of dental caries (ORmax = 1.30, 95% CI = 1.10–1.53, *P* = 0.026) (Table 3).

**Table 3:** Logistic regression analysis of factors related to the prevalence of dental caries.

	Univariate		Multivariate	
	cOR (95% CI)	*P*-value	aOR (95% CI)	*P*-value
Age	0.96(0.95-0.97)	<0.001	0.92(0.89-0.95)	<0.001
BMI	0.99(0.98-0.99)	0.001		
Sex				
Male	0.85(0.81-0.89)	<0.001	0.84(0.79-0.89)	<0.001
Female	1.00			
Areas				
Urban area	1.00		1.00	
Rural area	2.63(2.47-2.79)	<0.001	2.55(2.40-2.72)	<0.001
Education stages		<0.001		<0.001
Primary school 4–6	1.23(1.14-1.31)	<0.001	1.25(1.12-1.40)	<0.001
Junior high school	1.21(1.13-1.30)	<0.001	1.41(1.14-1.73)	0.001
Senior high school	1.00		1.00	
Boarding school				
Yes	1.29(1.19-1.39)	<0.001	1.15(1.05-1.26)	0.003
No	1.00		1.00	
Drinking sugary beverages (past 7 days)		0.001		<0.001
Never	1.00		1.00	
Less than 1 time (per day)	1.09(1.02-1.15)	0.008	1.13(1.01-1.21)	<0.001
1 or more times (per day)	1.22(1.08-1.38)	0.001	1.23(1.07-1.42)	0.004
Eating fried food (past 7 days)		0.001		0.068
Never	1.00		1.00	
Less than 1 time per day	0.99(0.93-1.06)	0.782	0.95(0.82-1.02)	0.260
1 or more times per day	1.29(1.12-1.48)	0.001	1.13(0.96-1.32)	0.147
Eating fresh fruit (past 7 days)		0.032		0.006
Never	1.00		1.00	
Less than 1 time per day	1.22(1.03-1.45)	0.023	1.22(1.01-1.47)	0.037
Once a day	1.15(0.98-1.36)	0.090	1.29(1.07-1.54)	0.006
2 or more times per day	1.22(1.04-1.44)	0.017	1.35(1.12-1.62)	0.001
Eating vegetables (past 7 days)		<0.001		0.062
Never	1.00		1.00	
Less than 1 time per day	1.11(0.88-1.40)	0.393	1.04(0.81-1.34)	0.756
Once a day	0.98(0.79-1.22)	0.836	0.97(0.77-1.23)	0.813
2 or more times per day	0.86(0.69-1.06)	0.153	0.88(0.69-1.11)	0.296
Having breakfast (past 7 days)		<0.001		<0.001
Every day	1.00		1.00	
Occasionally	1.29(1.19-1.39)	<0.001	1.19(1.10-1.29)	<0.001
Never	1.20(1.02-1.41)	0.028	1.22(1.05-1.42)	0.048
Tooth brushing habit		0.001		0.038
Both morning and evening	1.00		1.00	
Only morning	1.16(1.06-1.28)	0.001	1.16(1.06-1.27)	0.041
Only evening	0.97(0.88-1.08)	0.605	1.03(0.93-1.14)	0.592
Occasionally or never	1.25(1.04-1.49)	0.015	1.14(0.95-1.37)	0.172
Using fluoride toothpaste		<0.001		<0.001
Yes	1.00		1.00	
No	1.14(1.04-1.24)	0.004	1.14(1.07-1.22)	<0.001
Do not know	1.15(1.08-1.22)	<0.001	1.09(0.99-1.19)	0.079
Average daily sleep duration		<0.001		0.036
< 8 h	1.00		1.00	
8–10 h	1.18(1.11-1.25)	<0.001	1.09(1.03-1.18)	0.034
> 10 h	1.45(1.19-1.78)	<0.001	1.30(1.10-1.53)	0.026

cOR is crude Odds Ratio. It is the OR value calculated from a univariate logistic regression analysis without covariate adjustment.

aOR is the adjusted Odds Ratio. It is the OR value calculated from a multivariate logistic regression analysis adjusted for other covariates.

Regarding brushing habits, a higher risk of caries was observed in participants who brushed only in the morning (OR = 1.16, 95% CI = 1.06–1.27, P = 0.041) compared to those who brushed in the morning and evening. Brushing solely at night was not significantly associated with an increased caries risk (*P* = 0.592). Additionally, not using fluoride toothpaste was linked to a higher risk of dental caries (OR = 1.14, 95% CI = 1.07–1.22, P < 0.001) (Table 3).

## Discussion

This study provided cross-sectional data on the prevalence and characteristics of dental caries among children and adolescents in Beijing. We also investigated the factors influencing dental caries among the study participants. The main finding was that lower grade levels, residing in a boarding school, coming from a rural area, consuming sugary beverages and fresh fruits, eating breakfast irregularly, inadequate teeth brushing, and not using fluoride toothpaste were positively associated with dental caries occurrence.

In terms of the baseline characteristics of the participants, we found that age, BMI, and gender are all associated with the risk of dental caries. A previous study by Tinanoff et al. highlighted that a child’s age was frequently utilized as an indicator to forecast their risk of dental caries, with older children generally exhibiting more severe caries [14]. Contrarily, in children aged 7–9 years, Liang et al. observed a potential decline in dental caries prevalence as age increased, attributed to the loss of primary teeth [15]. The outcomes of our research also support this view. The difference in outcomes could be because our study was carried out in the highly developed area of Beijing, and the study exclusively focused on student participants. With the advancement in age and educational attainment, participants likely gain enhanced understanding from various sources about the importance of preventing dental caries, thereby actively adopting relevant measures to mitigate their risk of developing dental caries. Regarding gender factor, Faris et al. reported no significant differences in the prevalence of dental caries between sexes [16]. In contrast, Chu et al. reported that girls had a significantly higher OR (1.14) for dental caries than boys [17]. Our study corroborates this observation, suggesting a lower prevalence of dental caries in boys than girls. This variation can be explained by the earlier tooth eruption in girls, which results in prolonged exposure to a cariogenic oral environment [18]. Additionally, a 2017 study by Ashour et al. identified a positive correlation between high BMI and caries [19]. In contrast, research by Bhayat et al. revealed a negative association [20]. However, studies by Ashi et al. showed a nonsignificant association between BMI and dental caries [21]. Consistent with the latter, our study findings revealed that although students with dental caries generally had a lower BMI compared to those without caries, no significant correlation between BMI and dental caries was established upon conducting a multivariate regression.

Dietary factors represented a crucial element in analyzing risk factors for dental caries. This study is the first to investigate the potential association between breakfast skipping and the higher risk of dental caries in the Beijing population. Similar to our research, Naimeh et al. reported that the DMFT score was 1.19 times higher in participants who irregularly consumed breakfast compared to those who regularly ate breakfast [22]. Several factors may contribute to children skipping breakfast, including the absence of available meals in the morning, the lack of meal providers, or inadequate education regarding the importance of breakfast. These factors are often linked to socioeconomic status (SES). Previous research indicated that children from lower SES are more likely to acquire dental caries [23]. As an SES indicator, coming from a rural area increased the risk of dental caries in this study. Fried foods are indeed processed foods and contain flavorings, which might be one of the aspects influencing dental health. A previous study indicated that consuming fried high-carbohydrate foods between regular meals can increase the presence of dentobacterial plaque [24], and in turn, this can harm the teeth and gums if exposed for an extended period of time [25]. This study did not identify a statistically significant association between the consumption of fried foods and the risk of dental caries; however, a potential trend of association was observed. In terms of fruit, according to Moynihan et al., fruit did not seem to contribute significantly to dental caries development [26]. However, research by Ruby and Love et al. presented a contrasting view, associating diets rich in sugar-laden fruits and berries with an increased caries incidence [27,28]. Our findings align with this latter observation, highlighting an increased prevalence of dental caries with fresh fruit consumption. For sugar-sweetened beverages, every additional serving of SSB consumed daily increased caries experiences by 22 percent [29]. According to Valenzuela et al., moderate SSB consumers had 1.22 more decayed, missed, or filled teeth than never/low-level consumers [30]. Valenzuela et al. identified that there is not only a positive correlation between SSB consumption and caries but also a dose-response gradient between them [30]. A significant increase in the risk of both caries (OR = 1.57) and erosion (OR = 1.43) was observed when comparing moderate-to-low consumption. An additional increase in caries (OR = 1.53) and erosion (OR = 3.09) was observed when high consumption was compared with moderate consumption [30]. The results of these studies are consistent with our findings: sugar-sweetened beverages were positively associated with the development of dental caries. The consumption of free sugars, encompassing added sugars in food and drinks, as well as naturally occurring sugars in syrups, fruit juices, and fruit juice concentrates, plays a significant role in the emergence of dental caries [31].

Our results indicated a positive correlation between sleeping for extended periods beyond 8 hours and the risk of dental caries. Conversely, a previous Japanese study found that children with shorter sleep durations of 8–9 hours per night had more caries than those with longer sleep durations over 9 hours [32]. The difference may be because our study participants varied in age. The Japanese research focused on participants aged 6–12, while the average age of our study participants was 12.80 ± 2.51. In addition, our study participants were limited to the student population. Due to academic pressures, many students did not get adequate sleep, with those sleeping more than 10 hours constituting a small proportion. Another study aligns with our results: Moro et al. found that long sleep duration, often more than 9 hours nightly, correlated with an increased risk of dental caries in children [33]. So, a possible explanation is that longer sleep duration can alter the regular flow of saliva, causing shifts in the makeup of oral microbes and elevating the risk of dental caries [34]. Our data indicate that brushing only in the morning or evening, compared to brushing both morning and evening, increased the risk of developing cavities. The findings of Nguyen et al. are consistent with our results, suggesting that brushing twice daily is an effective self-care strategy for caries prevention and tooth preservation [35]. Moreover, we found that the impact of brushing at different times of the day appears to vary: brushing only in the morning was associated with a higher risk of dental health, but for those who only brush their teeth at night, the risk of cavities did not show a significant increase. Previous studies also revealed a negative correlation between evening toothbrushing frequency and the number of new carious lesions [36]. Meanwhile, the frequency of morning toothbrushing showed no relation to cavity development, except for teeth needing crown restoration or extraction [36]. Our study believes this result may be tied to their daily habits. When sugary or starchy foods are consumed, bacteria in the mouth break down the carbohydrates and produce lactic acid [37]. This acid decreases the oral pH, leading to the development of dental caries [37]. Individuals who brush their teeth in the evening typically have a shorter period before going to bed, reducing the likelihood of consuming food. In contrast, those who only brush in the morning have the entire day ahead, during which food intake is likely significantly more significant than the amount consumed between evening brushing and bedtime. Hence, we infer that this might be one of the reasons why there is no marked increase in the risk of cavities for those who brush only at night. Another critical element in the discussion of toothbrushing was the utilization of fluoride toothpaste. Supported by more than half a century of research, the merits of fluoride toothpaste in preventing dental caries have been firmly entrenched. A study from the Nepali region reinforced this assertion, highlighting the significant reduction in caries, primarily due to the widespread adoption of fluoride toothpaste among schoolchildren aged 12 and 13 [38]. Our study also confirmed that not using fluoride toothpaste is a risk factor for cavities. This finding might be explained by the role of fluoride toothpaste in reducing caries by targeting the tooth surface and reducing its sensitivity to acid attacks [39]. Fluoride toothpaste halts caries progression, diminishes demineralization, and enhances the remineralization of already demineralized tissue [39].

The major strength of this study lies in its large sample from different school stages in the capital city, supported by detailed and accurate records from a government-led health monitoring project, which played a significant role in enhancing teeth protection. There are also some limitations: First, the results of this study were well representative of children from regular schools in Beijing, but there may be limitations to the extrapolation of the findings, especially in the representation of the Chinese child population. Since our study did not include children from special schools, such as those for children with severe physical or psychological conditions or impairments, this may affect the generalizability of the results. Furthermore, the study did not include an analysis of health-related factors of dental caries, such as regular caries check-ups or the use of dental floss. Data on family and parental socio-economic were unavailable. Since the survey questionnaire relies on student self-reporting, memory bias was unavoidable. Moreover, although the questionnaire was developed based on established literature and expert input, and has been consistently applied in Beijing for many years, additional rigorous validation remains warranted to establish its validity thoroughly. Additionally, as this study involved a large sample size, we may find several mathematically significant factors that may not be clinically significant. It is necessary to improve in future epidemiological studies. Last, research on the oral health status of children in the Beijing area may require longitudinal studies to explore trends to guide the development of interventions. Therefore, well-designed cohort studies will be needed for future explorations in this domain.

## Conclusions

In this study, dental caries was a prominent issue among children and adolescents in Beijing area. Factors such as residing in boarding school, coming from a rural area, frequent consumption of sugary beverages, fruit consumption, breakfast irregularity, infrequent tooth brushing, and longer sleep durations are associated with a high prevalence of dental caries. It is recommended that oral health education for children and adolescents in Beijing be reinforced to facilitate the development of positive oral health habits and thereby reduce the prevalence of dental caries.

## Supporting information

S1 Fig The flow chart shows a multi-stage stratified random cluster sampling technique of the present study. (TIF)

S1 Appendix Questionnaire on Students’ Health Status and Influencing Factors. (TIF)

## Abbreviations

DMFT: Decayed, Missing, and Filled Teeth (permanent teeth)
dmft: decayed, missing, and filled teeth (primary teeth)
CI: Confidence Interval
OR: Odds ratios
cOR: Crude odds ratios
aOR: Adjusted odds ratios
H: Hour
WHO: World Health Organization
SSB: Sugar-sweetened beverage
SES: Socioeconomic status
CPI probe: Community periodontal index probe.
