# Supplementary figures and images for: Prevalence of dental caries and influence factors among students in Beijing: A cross-sectional study
Source: PLoS One. 2025 Apr 29;20(4):e0322694. doi: 10.1371/journal.pone.0322694 (PMC12040134; doi:10.1371/journal.pone.0322694)

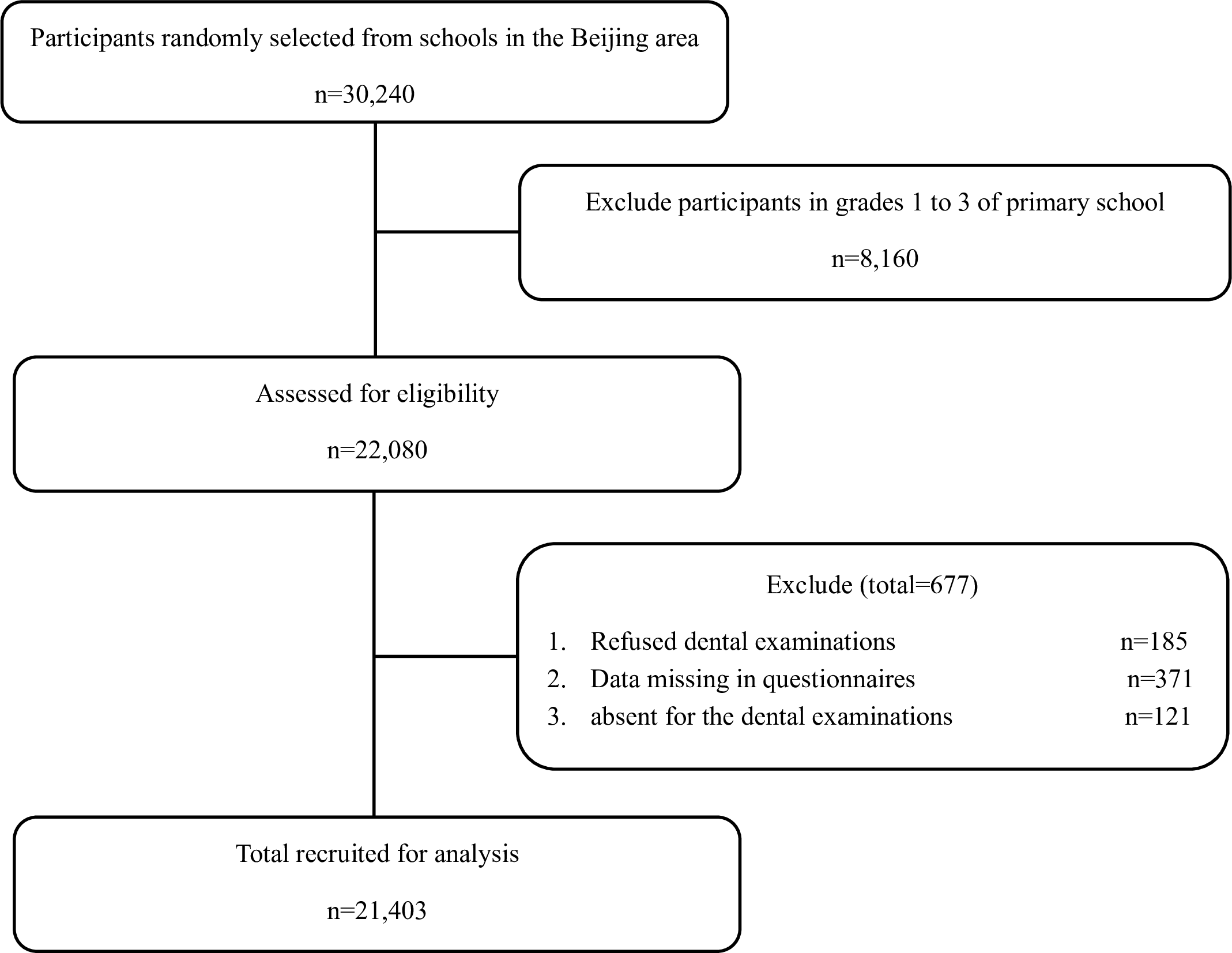

Supplement: S1 Fig — (TIF) [file pone.0322694.s001.tif]

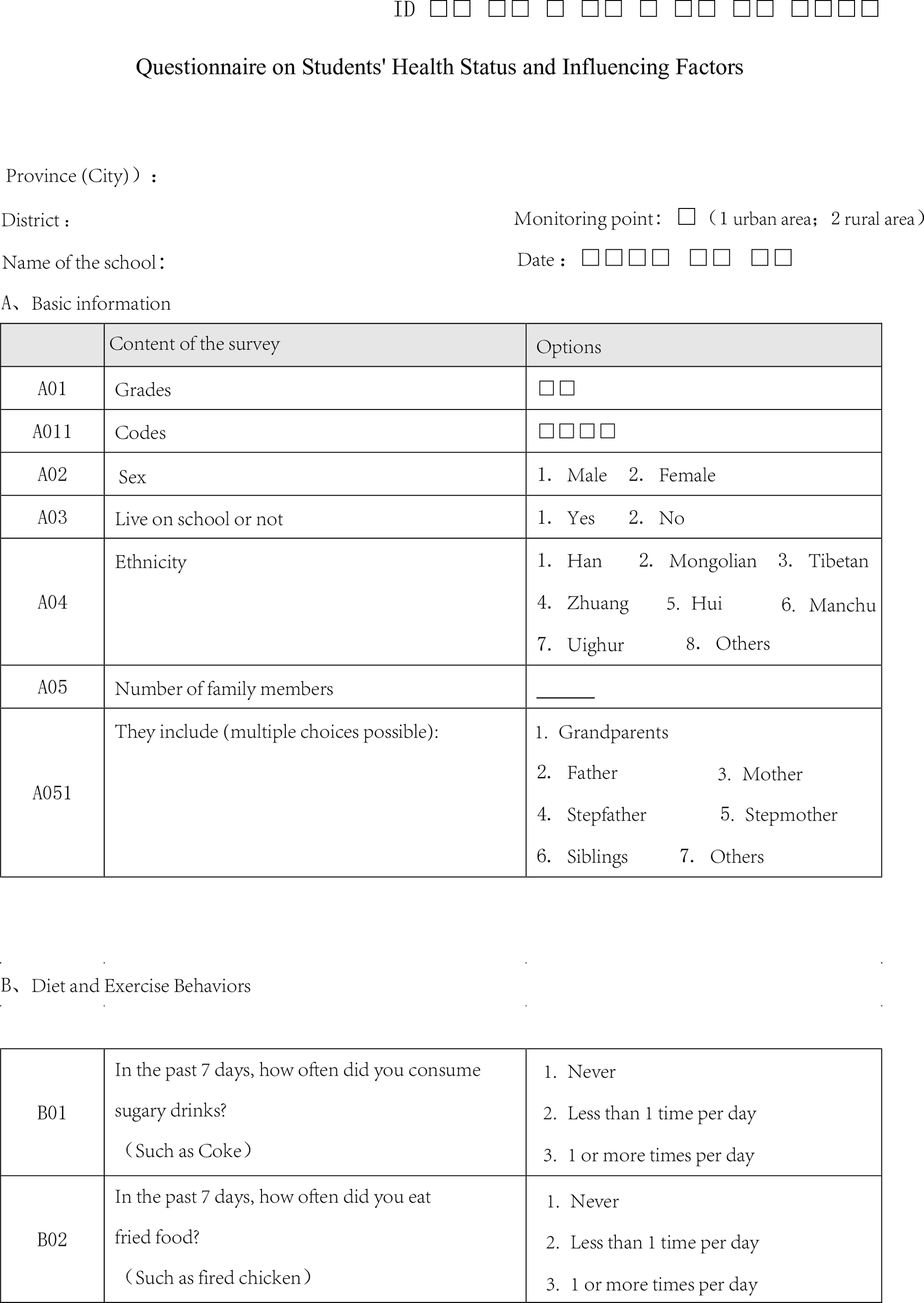

Supplement: S1 Appendix — (TIF) [file pone.0322694.s002.tif]
